# Neuroplastin 65 deficiency leads to the impairment of visual function through affecting ribbon synapse in retina of mice

**DOI:** 10.3389/fncel.2025.1558334

**Published:** 2025-05-08

**Authors:** Jiu-jiang Zeng, Ling Chen, Li-fen Liu, Jia-lu Wang, Jie Cheng, Ya-ni Zheng, Lei Zhang, Xiao-ming Zhang, Qiong-lan Yuan

**Affiliations:** ^1^Department of Neurology, Shanghai Tongji Hospital, Tongji University School of Medicine, Shanghai, China; ^2^Department of Human Anatomy, Histology and Embryology, Tongji University School of Medicine, Shanghai, China; ^3^Department of Human Anatomy, Jinggansan University School of Medicine, Jian, China

**Keywords:** neuroplastin 65, visual function, ribbon synapse, ribeye, retina

## Abstract

Neuroplastin 65 (NP65) is a synapse-enriched glycoprotein in the central nervous system and is implicated in synaptic plasticity. In the present study, we found that NP65 knockout (NP65 KO) mice exhibit impaired visual function, including reductions in the amplitude of b-wave in scotopic flash electroretinogram (fERG), the amplitude of N1 and P1 waves in flash visual evoked potentials (fVEP), and the constriction rate in pupillary light reflexes (PLR). In wild-type (WT) mice, NP65 is specifically enriched in the synaptic ribbon (SR) of ribbon synapses labeled by Ribeye in the retina. We found that NP65 KO mice display nearly normal architecture of the retina. However, NP65 KO mice show a significant decrease in the immunoreactivity of presynaptic postsynaptic density protein 95 (PSD95), synaptophysin (SYN) and Ribeye in the outer plexiform layer (OPL). Moreover, the electron microscopy displays a decrease in synaptic ribbons and defects in postsynaptic structures in the ribbon synapses of the OPL in NP65 KO mice. In addition, we found that the apposition of presynaptic photoreceptor axonal terminals and postsynaptic bipolar cell dendrites in the OPL is misplaced in NP65 KO mice. Finally, we show that intravitreous injection of AAV-NP65 reverses the visual dysfunction, increases Ribeye expression and restores the normal arrangement in the OPL of NP65 KO mice. Together, our findings reveal that NP65 deficiency leads to visual function impairment by affecting ribbon synapses in the OPL of mice, suggesting that NP65 is critical for visual function in mammals and a potential target for degenerative retinopathy.

## Introduction

Neuroplastins are neuronal and synapse-enriched glycoproteins belonging to the immunoglobulin (Ig) superfamily of neural cell adhesion molecules (NCAMs) (Beesley et al., 2014). The Nptn gene transcript produces two Neuroplastin (NP) protein isoforms, NP65 and NP55, named according to their molecular weight. Both isoforms contain a short intracellular C-terminal domain, a transmembrane domain, and extracellular Ig modules, and are distinguished solely by the presence of the Ig1 module in NP65 and its absence in NP55 (Hill et al., 1988; Langnaese et al., 1997). NP55 is widely expressed in various tissues, such as the liver and kidney, whereas NP65 is mainly distributed in the brain and retina (Bernstein et al., 2007; Smalla et al., 2000; Kreutz et al., 2001). Increasing evidence shows that NP65 is implicated in hippocampal synaptic plasticity and neuronal plasticity (Smalla et al., 2000; Empson et al., 2006; Owczarek et al., 2011) likely through direct interaction with the amino-terminal domain (ATD) of GluA1 (Jiang et al., 2021) and NP65 homophilic interactions (Smalla et al., 2000; Owczarek et al., 2011).

Notably, a series of reports have shown that NP65 is related to mental and cognitive disorders as well as ischemic brain injury using NP65-deficient mice (Amuti et al., 2016; Cheng et al., 2024; Hu et al., 2017; Li et al., 2019). For example, NP65-deficient mice have been reported to display anxiety-like behaviors and enhanced spatial learning and memory (Amuti et al., 2016; Li et al., 2018; Li et al., 2019). In addition, NP65 deficiency has been shown to potentiate neuronal loss in mouse ischemic brain injury (Hu et al., 2017). All of these studies uncover the crucial role of NP65 in brain functions in rodents. Although reports have shown that NP65 is restricted to the outer plexiform layer (OPL) and inner plexiform layer (IPL) of the rat retina (Kreutz et al., 2001), whether NP65 is required for visual function is still undetermined.

The eye, as a special sensory organ, is supremely well adapted for detecting input from the external environment. The retina, as a part of the central nervous system, is characterized by a distinct laminar organization where several types of cells communicate through conventional and ribbon synapses to codify visual information (Hoon et al., 2014). The complex neuronal network of the retina consists of five major classes of neurons (photoreceptor cells, bipolar cells, horizontal cells, amacrine cells, and ganglion cells), building up ten layers of the retina. Retinal synaptic contacts are developed within two distinct layers, OPL and IPL (Furukawa et al., 2020). Ribbon synapses are specially located in the OPL and IPL of the vertebrate retina, where they are best characterized in sensory synapses with extraordinary signaling requirements (Schmitz et al., 2000). In the OPL, the photoreceptor ribbon synapse has a unique triad structure with bipolar and horizontal cell processes invaginated into photoreceptor terminal (Burger et al., 2021). In addition, the horizontal cell processes form inhibitory contact with photoreceptor terminals and bipolar cell dendrites, mediating feedback and feedforward regulation of the synaptic transmission between photoreceptor and bipolar cells, respectively (Diamond, 2017). In the IPL, bipolar cells form ribbon synapses with amacrine cells and ganglion cells. Given that NP65 is highly localized in the OPL and IPL and the majority of NP65 immunoreactivity is of peri-synaptic origin (Kreutz et al., 2001), it is important to determine whether NP65 is crucial for visual function.

In the present study, we utilized the NP65 deficiency mouse model (Amuti et al., 2016) to investigate the role of NP65 in visual function. Importantly, our study provide evidence that the synaptic protein NP65 is required for normal visual function in mice.

## Materials and methods

### Animals

The NP65 knock-out (NP65 KO) mice were obtained as described previously (Amuti et al., 2016). Wild-type C57BL/6 mice (WT mice) from the same background strain were used as controls in all experiments. All animals were housed on a 12 h light-dark cycle in a temperature- and humidity-controlled environment with access to water and food *ad libitum*. The experiments were approved by the Tongji University Animal Care Committee.

### Visual electrophysiological tests of NP65 KO mice

To determine whether NP65 deficiency affects visual function, a series of visual responses including pupillary light reflexes (PLR), flash visual evoked potentials (fVEP), and scotopic flash electroretinogram (fERG) were successively performed by a blinded investigator using a cohort of adult NP65 KO and WT mice (2 months old). First, PLR was measured in WT (*n* = 8) and NP65 KO (*n* = 8) mice according to the previous reports (Jain et al., 2016; Sun et al., 2014; Xue et al., 2011). Briefly, mice were dark adapted for 30 min, then lightly anesthetized with pentobarbital sodium (0.1 mg/10 g) in a dark room. The animals were then positioned on a test platform and the right eyes were completely covered with silver paper while the direct pupil response of the left eye was measured. Consensual PLR was recorded using an infrared video camera (frame rate 25 Hz, PC164CEX-2; Supercircuits Inc., Austin, TX, United States), which sent the image to a computer. A high-power light-emitting diode (MCWHL2/white, M625L2/red, M470L2/blue; Thorlabs, Inc., Newton, NJ, United States) was set to provide illumination for this infrared camera. Following the baseline recording for 60 s in the dark, the PLR of all mice was recorded for 5 s at high irradiance (2.9 × 10^4^ μW/cm^2^). This response was repeated for 4-6 times per animal. For stop-frame analysis, the computer recorded the data at a rate of 3 frames per second. The recorded responses were replayed on the video frame by frame, and pupil images were analyzed using batch processing in Photoshop (CS6 extended; Adobe Systems, Inc., San Jose, CA). The maximum diameter before light stimulation (baseline diameter) and the minimum diameter after stimulation, as well as the latency of pupillary constriction and the velocity and time of pupillary constriction of the pupillary light response, were analyzed.

On the 7th day after PLR, these mice were followed to be used for fVEP as described previously (Sun et al., 2014; Ridder and Nusinowitz, 2006; Tomiyama et al., 2016) with a small animal visual electrophysiological detection system (Ming Kang Sciences Co., Ltd., Chongqing, China). Briefly, after 30 min in a dark room, the mice were lightly anesthetized with an intraperitoneal injection of pentobarbital sodium (0.1 mg/10 g) and then placed in a stereotaxic frame (Alcott Biotect CO., Ltd., Shanghai, China). After making a cross-incision on the skin to expose the skull lambda, the skull was removed with hydrogen peroxide until the dura mater was exposed. A hole (0.5 mm in diameter) was then drilled in the dura mater over the right primary visual cortex (V1, 2.5 mm lateral to lambda) to expose the underlying pia mater and V1 cortex. An active (recording) electrode was placed on the surface of the visual cortex. A passive electrode was implanted over the right frontal areas (1 mm lateral and 2 mm anterior to bregma), and a grounding electrode was placed on the tail of the mouse. The illumination light in the experimental room was turned off, and the left eye of the mouse was covered with silver paper. The right eye of the mouse was stimulated with a single light flash (intensity 2.0 log cd.s/m^2^, 1.0 Hz frequency) emitted by a white light-emitting diode placed at 5 mm from the cornea. During all recordings, the mouse was kept on a heated pad to maintain a temperature of 37°C and recorded five times. The signals were amplified (gain × 1,000) and band-pass filtered (1,000 Hz) using a differential AC amplifier (A-M systems Micro Electrode amplifier model 1800). The signals were then digitally converted (Digidata 1440A, Axon Instruments) and analyzed with pClamp 10 (Molecular Device). The amplitude with respect to baseline and latency from stimulus onset of the N1 and P1 components of FVEPs were measured and analyzed.

On the 7th day after fVEP, these mice were used to perform fERG tests (Contreras et al., 2021; Mdzomba et al., 2018). After being dark-adapted overnight for at least 12 h, the mice were anesthetized in scotopic conditions with 0.07% pentobarbital sodium (0.1 mg/10 g). Their pupils were dilated with a drop of 0.25% tropicamide (Shuangke Drug Co., Ltd., Hefei, China), and 0.9% saline was applied to the cornea to prevent drying. The animals were positioned on a temperature-regulated platform and electrodes were placed on the cornea of each eye. Reference electrodes were subcutaneously placed on each side of the temporal area, and the ground electrode was placed on the ear-lobe. The animal’s body temperature was maintained at 37°C using a heating pad. Both eyes were recorded simultaneously using the visual electrophysiological APS-2000AER system (Ming Kang Sciences Co., Ltd., Chongqing, China). ERGs were obtained in response to a single white flash stimulus at a frequency of 0.5 Hz and an intensity of 2.0 log cd.s/m^2^. Responses were amplified at a gain of 10,000 at 0.1-300 Hz. For quantitative analysis, the amplitude of the a-wave was measured from the baseline to the most negative trough, while the b-wave was measured from the trough of the a-wave to the most positive peak of the retinal response. The latency of both waveforms was measured from the stimulus onset to the peak amplitude.

### Retinal preparation for histology

After being deeply anesthetized with pentobarbital sodium, mice were transcardially perfused with 0.9% saline followed by 4% paraformaldehyde in phosphate buffer (PB). After the optic nerve was cut, the superior part of the eye was marked with a felt pen, and the eye was removed as previously described (Koulen et al., 1998). For light microscopy, the eyes were opened along the ora serrata, and the eyecups were immersed in 4% paraformaldehyde for 2 h and rinsed in PB. After the vitreous body was removed, the eyecup (posterior part of the retina) was post-fixed in 4% paraformaldehyde at 4°C overnight. The retina was cryoprotected in 10, 20, and 30% sucrose in PB overnight at 4°C, and embedded in OCT compound. Consecutive vertical sections of the retina (10 μm thick) were prepared using a cryostat microtome (Leica CM1950, Wetzlar, Germany).

### Nissl staining

After washing with PBS, the retinal sections were incubated with Nissl Staining Solution (Beyotime Institute of Biotechnology, Shanghai, China) for 5 min at room temperature. The sections were then dehydrated with graded ethanol, vitrified with dimethylbenzene, and covered with neutral gum.

### Immunofluorescent staining for retina

The immunostaining protocols for retinal sections were described previously with minor modifications (Takada et al., 2008). In brief, to expose the antigen epitope, retinal sections were heated in a microwave oven until boiling in citrate buffer (pH 6.0). The heating process was stopped and the sections were allowed to cool for a while before repeating the process 5-7 times. The sections were then naturally cooled to the room temperature (RT). These sections were then permeabilized with 0.4% Triton X-100 in PBS for 30 min, and then blocked with 5% bovine serum albumin (BSA, Sigma, United States) for 60 min at RT. Next, the sections were incubated with primary antibodies (listed in Table 1) overnight at 4°C. After washing with PBS, the sections were incubated for 2 h at RT with appropriate Alexa Fluor 488-conjugated goat anti-mouse/rabbit IgG or Cy3-conjugated donkey anti-goat or goat anti-mouse/rabbit (Jackson ImmunoResearch, West Grove, PA, United States, dilution 1:1,000). Finally, after thorough washing, the sections were mounted with antifade mounting medium containing DAPI. The primary and secondary antibodies were diluted with 1% BSA in 0.3% Triton X-100 in PBS.

**TABLE 1:** Antibodies used in the study.

Antibody	Dilution	Company
Rabbit anti-neuronal nuclei antigen (NeuN)	1:1,000	Abcam, United Kingdom
Mouse anti-Ribeye/CtBP2	1:200	BD transduction laboratories, United States
Rabbit anti-synaptophysin (SYN)	1:100	Abcam, United Kingdom
Rabbit anti- protein kinase alpha (PKCα)	1:200	Abcam, United Kingdom
Mouse-anti vesicular glutamate Transporter 1 (VGLUT1)	1:200	Sigma, United States
Goat anti-NP65	1:200	R&D systems, United States
Goat anti-postsynaptic density protein 95(PSD95)	1:200	Abcam, United Kingdom
Rabbit anti-glial fibrillary acidic protein (GFAP)	1:1,000	Abcam, United Kingdom
Mouse anti-calbindin	1:200	Sigma, United States
Mouse anti-glutamine synthetase (GS)	1:500	BD Bioscience, United States
Rabbit anti- vesicular GABA/glycine Transporter	1:200	Abcam, United Kingdom
(VGAT)	1:200	Abcam, United Kingdom

For double labeling, retinal sections were incubated with the following antibody cocktails: NP65 with vesicular glutamate transporter 1 (VGLUT1), SYN, calbindin, ribeye or protein kinase alpha (PKCα); PSD95 with SYN, Ribeye, PKCα, or calbindin; calbindin with SYN or PKCα; PKCα with Ribeye at 4°C overnight. Finally, these sections were then incubated with appropriate Cy3-conjugated or Alexa Fluor 488-conjugated IgG and covered with Permount.

All images were captured under a 400 × magnification using a confocal microscope (Olympus FV1000, Tokyo, Japan) and analyzed with ImageJ software v1.51 (National Institutes of Health, Bethesda, MD, United States). The optical density (OD) of PSD95, SYN and Ribeye immunoreactivity were calculated as the mean gray level value in a selected ROI (35 × 16 pixels) in OPL. Three or four sections per mouse (one close to the posterior part of eyecup; two or three slices close to peripheral part of eyecup, *n* = 3/group) were used for statistical analysis the OD of PSD95, SYN and Ribeye immunoreactivity. The averaged OD of 3 selected ROIs was used as the OD of each mouse.

### Transmission electron microscopy (EM) of retina

For electron microscopy of the retina, NP65 KO and WT mice (2 months old, *n* = 3) were used. The mice were deeply anesthetized with intraperitoneal pentobarbital sodium, perfused with 0.9% saline followed by 4% paraformaldehyde (PFA) in PB, and their eyeballs were enucleated. Following removal of extraocular muscles and creating a small incision at the limbus of cornea, the eyeballs were fixed in 4% PFA at room temperature for 2 h. Subsequently, an encircling cut was made to open the eyeball, and the posterior eyecup was further fixed in 4% PFA overnight at 4°C. After three washes in PBS for 1 h each, the retina was sectioned into four patches and then into small pieces (1 mm × 2 mm) under a dissecting microscope. The small retinal pieces were pre-fixed in 2.5% glutaraldehyde in PB for 4 h at room temperature, followed by three rinses with PB (each for 10 min) on a shaker. Post-fixation was done in 1% osmic acid for 2 h at room temperature, followed by three rinses in PB, dehydration in a series of acetone concentrations (30, 50, 70 and 90%) for 30 min each, and final dehydration in 100% acetone three times for 10 min each. Next, the retinal pieces were successively immersed in EPON812 resin:acetone solutions with volumes of 3:7 and 7:3 at room temperature overnight. Subsequently, the retinal pieces underwent a 3 h immersion in pure EPON812 resin followed by embedding and polymerization in EPON812 resin at 60°C for 48 h. Ultrathin (70 nm) retina slices were obtained using a Leica UC-6 ultrathin slicer (Leica Microsystems, Germany) and mounted on a 200-mesh copper grid. The slices were stained with 3% uranium acetate for 20 min and 1% lead citrate for 10 min at room temperature. Transmission EM (JEM-1230, Japan) was utilized to observe the ultrathin sections at × 6,000 magnification with an accelerating voltage of 80 KeV.

Two slices per animal were analyzed. The optical density (OD) of the synaptic ribbon was determined by subtracting the mean gray value (MGV) of the cytoplasmic area adjacent to the Ribbon (MGV_ROI–background_) from the MGV of the Ribbon itself (MGV_ribbon_), expressed as OD_ribbon_ = MGV_ribbon_ − MGV_ROI–background_. The MGV of the designated region of interest (ROI) was quantified using ImageJ software v1.51 (National Institutes of Health, Bethesda, MD, United States). Fifteen ribbons from three mice in each experimental group were assessed and subjected to statically analysis.

### Western blot analysis

After the WT and NP65 KO mice (*n* = 4, 2 months old) were deeply euthanized with pentobarbital sodium, their eyes were rapidly removed and the retina was obtained by dissecting the eyes on ice under a microscope. The total proteins of the retina were extracted using RIPA buffer (Beyotime, Shanghai, China). After determining the protein concentrations, protein samples (15 μg/lane) were separated by sodium dodecyl sulfate polyacrylamide gel electrophoresis and transferred onto nitrocellulose blotting membranes. The nitrocellulose membranes were incubated with primary antibodies, including NP65 (1:1,000), NeuN (1:1,000), GS (1:500), VGAT (1:1,000), PKCα (1:1,000), and calbindin (1:1,000), as well as rabbit anti-GAPDH (1:1,000) overnight at 4°C. The membranes were then washed and incubated with anti-rabbit/goat IgG-HRP or anti-mouse IgG-HRP (1:1,000, Beyotime) for 2 h at room temperature. Immunoreactive bands were visualized and images were quantitatively analyzed using ImageJ software (NIH, Bethesda, United States). Protein levels were normalized to those of GAPDH from three independent experiments.

### Intravitreal injection of adeno-associated virus (AAV) expressing NP65

The vectors for overexpressing mouse NP65 were constructed and prepared as described previously (Cheng et al., 2024). Intravitreal injections of AAV-NP65 or AAV-CON were performed in the bilateral eyes of each mouse, as previously described (Takada et al., 2008; Mdzomba et al., 2018). In brief, WT and NP65 KO mice (*n* = 10/group, 8-10 weeks old) were intraperitoneally anesthetized with pentobarbital sodium (0.1 mg/10 g) and intramuscularly injected with xylazine hydrochloride (0.1 g/100 g body weight, Nanning Hua Nuo Veterinary Medicine Co., Ltd., China) to relax their muscles. The mice were placed under a stereomicroscope (Alcott Biotect CO., Ltd., Shanghai, China), and a No.30 gauge needle tip was inserted through the sclera into the dorsal quadrant of the eyeball, next to the limbus. A small amount of vitreous humor was then extracted, taking care not to injure the lens. Next, 2 μL of AAV-NP65 or AAV-CON (as a control) was intravitreally injected into the eye at a speed of 0.5 μL/min using a 5-μL Hamilton syringe. The needle tip was kept in place for 5 min after injection to allow for diffusion of the virus solution in the vitreous body, and then slowly withdrawn to prevent backflow. The site of injection was sealed with surgical glue. The animals were kept at 37°C after surgery until they had fully recovered. On the first day after the operation, all the animals exhibited normal activities and ate their regular diet.

Four weeks after viral injection, the mice treated with AAV-CON or AAV-NP65 were subjected to visual electrophysiological tests as mentioned above, and the retinas were collected for immunostaining.

### Statistical analysis

All statistical analyses were performed using SPSS statistics software v18 (IBM, Armonk, NY, United States). Data are expressed as means ± standard error of the mean (S.E.M). Unpaired Student’s *t*-tests were used to evaluate the difference between two groups. Multiple comparisons were performed using one-way analysis of variance followed by the Fisher’s least significant difference test if the data were normally distributed. Otherwise, the Mann-Whitney U test was used to determine the significance among multiple comparisons. Differences were considered statistically significant when the *P*-value was < 0.05.

## Results

### NP65 KO mice show impaired visual function

**FIGURE 1:**
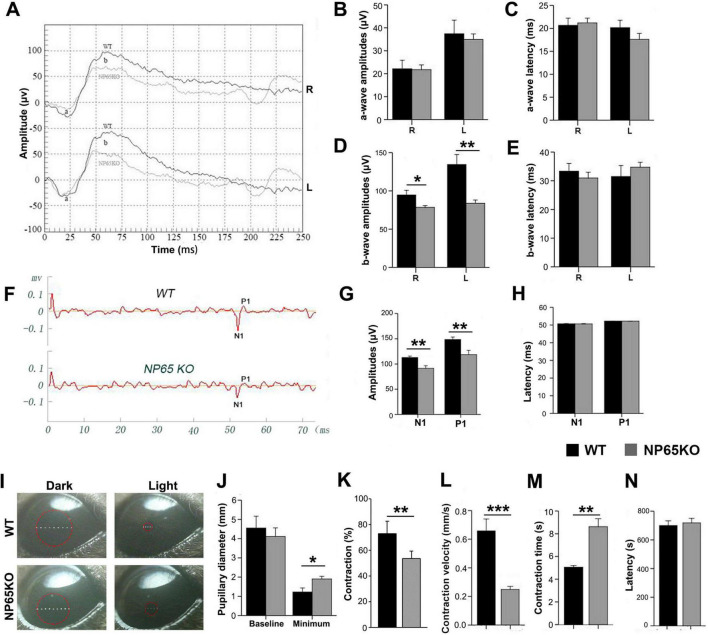
NP65 KO mice exhibit impaired visual function. **(A**–**E)** NP65 KO mice show severe deficits in the scotopic flash electroretinogram (fERG). **(A)** Representative fERG waveforms recorded from an NP65 KO and a WT mouse; **(B,C)** NP65 KO mice (*n* = 7) displayed similar amplitudes **(B)** and latencies **(C)** of a-wave compared with WT mice (*n* = 6); **(D,E)** NP65 KO mice showed a significant reduction in b-wave amplitude (**D**) while having an unchanged b-wave latency (**E**). **(F–H)** NP65 KO mice exhibit impairment in the flash visual evoked potentials (fVEP). **(F)** Representative fVEP waveforms from an NP65 KO and a WT mouse; **(G)** NP65 KO mice (*n* = 7) showed a significant decrease in the amplitude of N1 and P1 relative to WT mice (*n* = 6). **(H)** NP65 KO mice displayed comparable latencies of N1 and P1 compared with WT mice. **(I-N)** NP65 KO mice exhibit a reduced response in the pupillary light reflexes (PLR). **(I**) Representative pupil images from an NP65 KO and a WT mouse, a red circle placed on the eye indicating the size of the pupil; **(J)** NP65 KO mice (*n* = 8) had an unchanged baseline pupillary diameter while showing a larger minimum pupillary diameter compared with WT mice (*n* = 8); **(K,L)** NP65 KO mice exhibited reduction in constriction **(K)** and velocity **(L)** of the pupil; (**M**,**N**) NP65 KO mice showed a longer time (**M**) and a similar latency (**N**) of constriction. *P < 0.05, **P < 0.01, ***P < 0.001.

For NP65, a synaptic protein, that has been shown to be highly expressed in the OPL and IPL of the retina (Kreutz et al., 2001), we wondered whether NP65 is implicated in visual function as measured by fERG, fVEP and PLR using a NP65 deficiency mouse model, as we have previously reported (Amuti et al., 2016). As shown in Figure 1, fERG was measured and analyzed for both eyes of WT and NP65 KO mice (Figures 1A-E). The results showed that the amplitude (Figure 1A) and latency (Figures 1A-C) of the a-wave in dark-adapted ERGs were comparable between WT and NP65 KO mice. However, the amplitude of the dark-adapted ERG b-wave was significantly reduced in NP65 KO mice, while the latency of the ERG b-wave remained unchanged (Figures 1A,D,E). This suggests an impaired transmission in the OPL of NP65 KO mice. In addition, we investigated whether the visual pathway of NP65 KO mice was affected by measuring fVEP and PLR. The representative recordings of fVEP in WT and NP65 KO mice are shown in Figure 1F. The fVEP responses showed that NP65 KO mice had lower amplitudes of N1 and P1 waves, but no effect on the latency of N1 and P1 waves compared to WT mice (Figures 1F-H). Furthermore, PLR is an important clinical index for assessing the integrity of visual pathways and is a typical non-image-forming response (Contreras et al., 2021) that reflects retinal function. We found that NP65 KO mice exhibited a light-dependent pupillary constriction similar to WT mice (Figure 1I), but with reduced contraction and velocity of the pupil, and an increase in contraction time, while having a similar latency of contraction and baseline pupillary diameter compared with WT mice (Figures 1J-N). Together, these results show that NP65 is crucial for visual function in mice.

### NP65 is highly expressed throughout layers of the mouse retina

**FIGURE 2:**
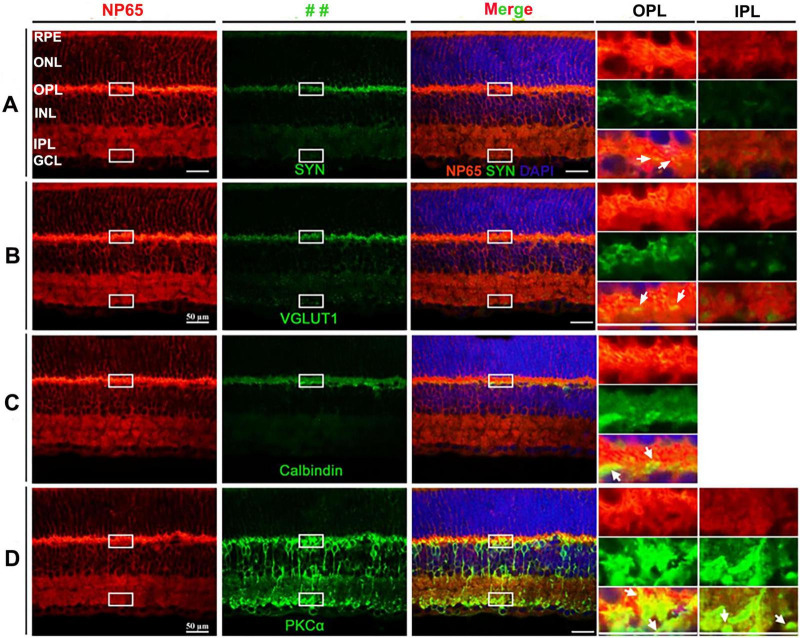
Distribution and cellular localization of NP65 in retina of WT mice. **(A–D)** NP65 was expressed throughout all layers of the retina. NP65 immunoreactivity was abundantly localized in the layers of the retina, including the RPE, ONL, OPL, INL, and IPL, and faintly in the GCL in the left panel of **(A–D)**. **(A,B)** Double immunostaining of NP65 with SYN **(A)** or VGLUT1 **(B)** showed co-localization of SYN or VGLUT1 with NP65 in the OPL and IPL. Insets: an enlargement of the white rectangle in the merge panel showing complete overlap (yellow, indicated by↑) of SYN or VGLUT1 with NP65 in the OPL and IPL. **(C**) Double immunostaining of NP65 and Calbindin in the retina. Insets: an enlargement of the white rectangle in the merge panel showing co-localization (yellow, indicated by↑) of NP65 and Calbindin in OPL. **(D)** Double immunostaining of NP65 and PKCα in the retina. Insets: an enlargement of the white rectangle in the merge panel showing co-localization of NP65 and PKCα in the inner boundary of the OPL and the outer boundary of the IPL. Representative images are shown and observed on three sections derived from WT mice (*n* = 3). RPE, retinal pigment epithelium; ONL, outer nuclear layer; OPL, outer plexiform layer; INL, inner nuclear layer; IPL, inner plexiform layer, GCL, ganglion cell layer; VGLUT1, vesicular glutamate transporter 1; SYN, synaptophysin; PKCα, protein kinase alpha. Scale bars = 50 μm.

**FIGURE 3:**
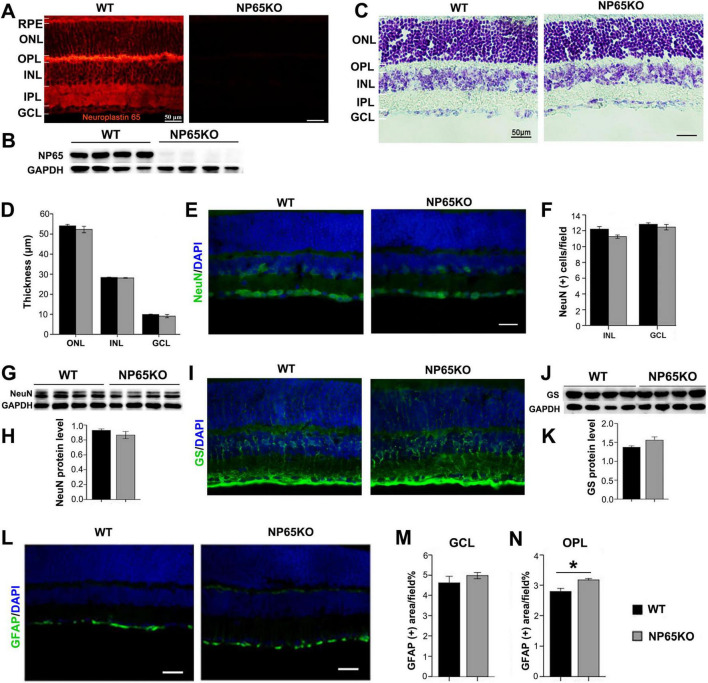
NP65 KO mice display normal architecture of the retina. **(A,B)** Immunostaining **(A)** and western blotting **(B)** showed a complete absence of NP65 in the retina of NP65 KO mice while abundant expression was observed in WT mice (*n* = 4). **(C,D)** Nissl staining showed that NP65 KO mice had an unchanged thickness of the retina compared with that of WT mice. **(E–H)** NP65 KO mice showed a comparable distribution and expression of NeuN in the retina compared with WT mic, as shown by immunostaining **(E,F)** and western blot **(G,H)**. **(I–K)** NP65 KO mice showed similar distribution and expression of Müller cells labeled with GS, as shown by immunostaining **(I)** and western blot **(J,K)**. **(L,N)** NP65 KO mice exhibited similar GFAP immunostaining in the GCL as WT mice and faint GFAP immunoreactivity in the OPL. Nuclei were stained with DAPI (blue). RPE, retinal pigment epithelium; ONL, outer nuclear layer; OPL, outer plexiform layer; INL, inner nuclear layer; IPL, inner plexiform layer; GCL, ganglion cell layer. GS, glutamine synthetase; GFAP, glial fibrillary acidic protein; NeuN, neuronal nuclei antigen. For staining statistical analysis, three sections per mouse were used from WT and NP65 KO mice (*n* = 5); for western blot statistical analysis, the data were from three independent experiments (*n* = 4). *P < 0.05, Scale bars = 50 μm.

To uncover the mechanism underlying the effect of NP65 on visual function, we investigated its distribution and cellular location in the mouse retina. In contrast with the previous reports which showed NP65 confined to the OPL and IPL in the retina (Kreutz et al., 2001; Carrott et al., 2016), we found that NP65 immunoreactivity was present in all layers of the retina, including the retinal pigment epithelium (RPE), outer nuclear layer (ONL), OPL, inner nuclear layer (INL), IPL and ganglion cell layer (GCL) (Figures 2, 3A). The most intense NP65 immunostaining was observed in the OPL, with moderate staining in the RPE, ONL, INL and IPL, and faint staining in the GCL (Figures 2, 3A). Notably, prominent immunostaining was also observed in the processes of the ONL and in the processes and cellular membranes of the INL (Figures 2, 3A). These results clearly show that NP65 is distributed throughout all layers of the retina and is highly expressed in the OPL, suggesting its potential role in synaptic transmission in the retina.

In addition, double immunostaining was used to investigate the cellular and subcellular localization of NP65 in the retina. Since VGLUT1 and SYN (synaptophysin) are known to be presynaptic structures and are expressed in the OPL and IPL of the retina (Romero-Aleman et al., 2010), the double immunostaining of NP65 with VGLUT1 or SYN showed that all immunofluorescence of VGLUT1 or SYN was completely colocalized with NP65 in the OPL and IPL (Figures 2A,B). This colocalization confirms that NP65 is localized in the photoreceptor axonal terminals (OPL) and bipolar cell axonal terminals (IPL), consistent with the previous report (Kreutz et al., 2001). Next, we observed whether NP65 is expressed in the horizontal and bipolar cells in the INL or not. As shown in Figure 2C, the double immunostaining of NP65 with calbindin for horizontal cells displayed that NP65/calbindin immunoreactive puncta were colocalized in the inner boundary of the OPL (Figure 2C, arrow indicated), confirming that NP65 is expressed in the processes of horizontal cells in the OPL. Besides, the double immunolabeling of NP65 with PKCα for bipolar cells showed that NP65 immunostaining overlapped with the dendrites of bipolar cells in the OPL and axon terminals of bipolar cells in the IPL (Figure 2D, arrow indicated). These observations demonstrate that NP65 is localized in both bipolar and horizontal cells. Taken together, all of these results show that NP65 is highly expressed in the retina, most abundantly in the OPL, and located in both the presynaptic and postsynaptic regions of the OPL and presynaptic region of the IPL.

### NP65 KO mice show unchanged architecture of retina

To explore the mechanisms underlying the impaired visual function in NP65 KO mice, we observed the retinal structures of NP65 KO mice. As shown in Figure 3, NP65 was completely absent in the retina of NP65 KO mice while it was abundant in the retina of WT mice similar to Figures 2, 3A,B. Nissl staining showed that NP65 KO mice displayed a normal laminar structure of the retina with an unchanged thickness (Figures 3C,D). In addition, NP65 KO mice had a comparable distribution and expression of NeuN (a neuronal marker) in the retina compared with WT mice, as measured by immunostaining and western blot analyses (Figures 3E-H), indicative of a normal inner half of the retina in NP65 KO mice. Both the NP65 KO and WT mice displayed a similar distribution and expression of Müller cells labeled with GS antibody, as shown by immunostaining and western blot analyses, respectively (Figures 3I-K). Lastly, GFAP-positive labeling of astrocytes was similar in the GCL of NP65 KO and WT mice, while faint GFAP immunoreactivity was found in the OPL of NP65 KO mice (Figures 3L,N). Together, NP65 KO mice show nearly normal retinal architecture with the exception GFAP staining in the OPL.

### NP65 KO mice show a decrease in synaptic ribbons of OPL in retina

**FIGURE 4:**
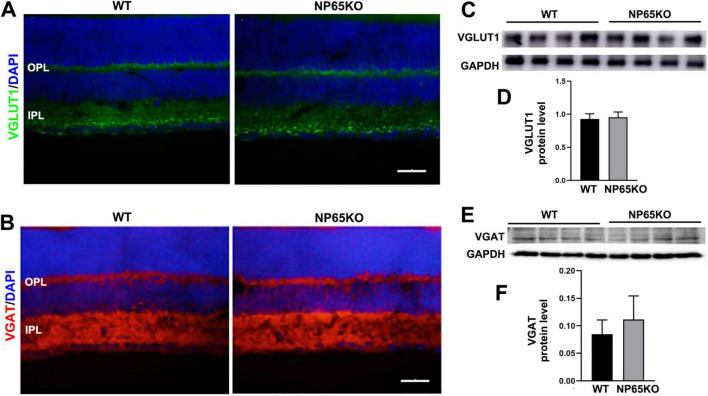
NP65 KO mice show normal level of excitatory VGLUT1 and inhibitory VGAT in retina. **(A)** NP65 KO mice displayed a similar distribution and immunoreactivity of VGLUT1 immunostaining in the OPL and IPL as WT mice (from three sections per mouse, *n* = 3). **(B)** NP65 KO mice had unchanged VGAT-positive structures in the inner half of the retina compared with WT mice (from three sections per mouse, *n* = 3). **(C–F)** Western blotting analysis showed that NP65 KO mice had comparable levels of VGLUT1 **(C,D)** and VGAT **(E,F)** in the retina relative to WT mice from three independent experiments (*n* = 4). VGLUT1, vesicular glutamate transporter 1; VGAT, vesicular GABA/glycine transporter. Scale bars = 50 μm.

The decrease in b-wave amplitude of fERG in NP65 KO mice suggests that synaptic transmission from photoreceptors to bipolar cells in the OPL may be impaired (Takada et al., 2008). Thus, we explored the neurotransmitters and presynaptic structures in the OPL of NP65 KO mice. VGLUT1, an indirect marker for glutamate level, is known to be expressed in the OPL and IPL as presynaptic terminals (Gong et al., 2006; Johnson et al., 2003; Singh et al., 2014). As shown in Figures 2B, 4A, VGLUT1 immunostaining formed continuous plexuses in the OPL and discrete punctate structures in the inner part of the IPL in WT mice, consistent with previous reports (Gong et al., 2006; Johnson et al., 2003). NP65 KO mice displayed a similar distribution and immunoreactivity of VGLUT1 immunostaining as WT mice (Figure 4A). In addition, VGAT, a marker for the inhibitory GABA transmitter, was observed in NP65 KO mice. As previously reported (Johnson et al., 2003; Kao et al., 2004), WT mice showed an intensive continuous plexus in the OPL and IPL and intense signals in the GCL (Figure 4B). Meanwhile, NP65 KO mice displayed similar VGAT-positive structures in the retina compared with WT mice (Figure 4B). Furthermore, western blotting analysis showed that NP65 KO mice had unchanged levels of VGLUT1 and VGAT in the retina relative to WT mice (Figures 4C-F). Therefore, these data suggest that NP65 deficiency has no effect on excitatory and inhibitory transmitter levels in the retina.

**FIGURE 5:**
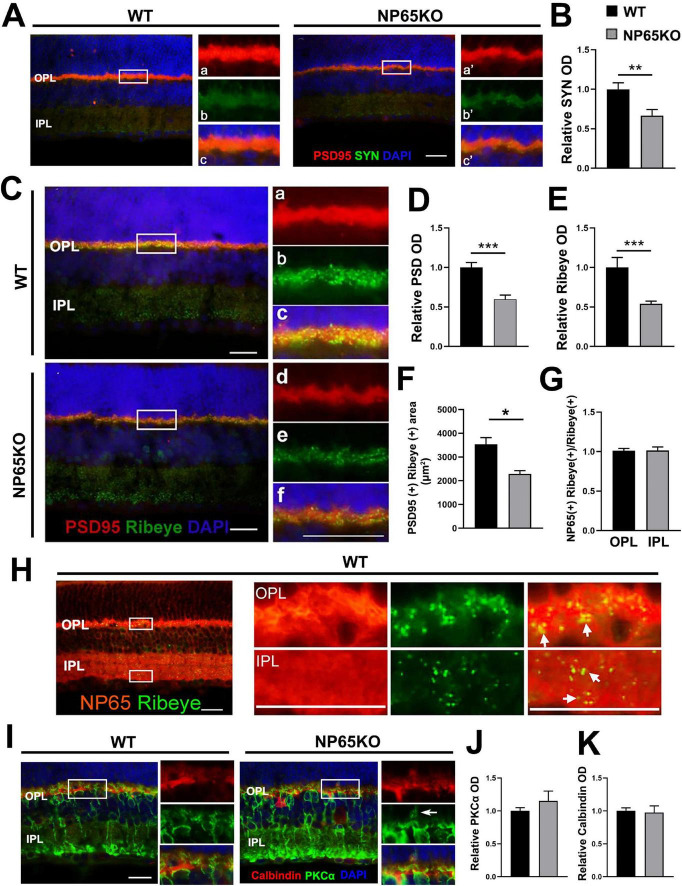
NP65 KO mice show a decrease in pre-synaptic PSD95, SYN and Ribeye in OPL. **(A-C)** Double immunostaining of PSD95/SYN **(A)** showed that NP65 KO mice had an unaltered distribution and co-localization of PSD95/SYN in the OPL. Quantitative analysis showed a significant reduction in optical density (OD) of SYN **(B)** immunoreactivity compared with WT mice. **(C–F)** Double immunostaining of Ribeye/PSD95 **(C)** showed that NP65 KO mice had a similar distribution of Ribeye-immunoreactive puncta in the OPL and IPL, and Ribeye co-localized with PSD95 as in WT mice **(C)**. Quantitative analysis **(D–F)** displayed a significant reduction in optical density (OD) of Ribeye and PSD95 immunoreactivity in the OPL of NP65 KO mice **(D,E)**; colocalization of Ribeye (+) with PSD95(+) was significantly decreased in the OPL of NP65 KO mice **(F)**. **(G,H)** Double immunostaining of Ribeye with NP65 showed that all Ribeye-positive puncta completely colocalized with NP65-positive structures in the OPL and IPL of WT mice. The nucleus was stained with DAPI (blue). The left panel: an enlargement of the white rectangle, which is notable for the large Ribeye-positive puncta in the OPL. **(I,J)** Immunostaining of PKCα displayed that NP65 KO mice had a slight increase in bipolar cell dendrites (**I**, arrows indicated), but had no significant difference in immunoreactivity **(J)** of the OPL compared with WT mice. **(I,K)** Immunostaining of calbindin showed that NP65 KO mice had a similar distribution **(I)** and comparable immunoreactivity **(K)** in OPL compared with WT mice. SYN, synaptophysin; PSD95, postsynaptic density protein 95; NP65, neuroplastin 65; OPL, outer plexiform layer; IPL, inner plexiform layer. For staining statistical analysis, three sections per mouse were used from WT and NP65 KO mice (*n* = 5). *P < 0.05; **P < 0.01; ***P < 0.001, Scale bars = 50 μm.

The presynaptic structures in the OPL represent the axon terminals of photoreceptors. SYN and PSD95 are known markers for the axon terminals of photoreceptors and conventional synapses in the retina (Takada et al., 2008; Koulen et al., 1998). Thus, the immunoreactivity of PSD95 and SYN was observed in the OPL. As previously reported (Koulen et al., 1998), PSD95 immunofluorescence was strongly prominent and colocalized with moderate SYN-immunoreactivity in the OPL of WT mice. NP65 KO mice showed similar PSD95 and SYN distribution characteristics to WT mice. However, the immunoreactivity of PSD and SYN was significantly decreased in NP65 KO mice (Figures 5A-D). Furthermore, Ribbon synapse is specific synapse in the OPL and IPL of the retina. Ribeye, a specific marker for synaptic ribbon in the retina (Schmitz et al., 2000; Maxeiner et al., 2016; Lv et al., 2016), was investigated in NP65 KO mice. Ribeye immunoreactivity was punctate and prominent in the OPL and moderate in the IPL, and overlapped with part of the PSD95-positive structures in WT mice (Figure 5C), as in previous reports (Schmitz et al., 2000; Maxeiner et al., 2016; Lv et al., 2016). Notably, Ribeye immunoreactivity and colocalization of Ribeye (+) with PSD95(+) were significantly decreased in the OPL of NP65 KO mice compared to WT mice (Figures 5C,E,F), indicating a diminution of synaptic ribbons in the OPL of NP65 KO mice. Furthermore, to explore whether NP65 is associated with Ribeye in the retina, double immunolabeling of NP65 and Ribeye was performed and revealed that all Ribeye-positive puncta were completely colocalized with NP65-positive structures in the OPL and IPL (Figures 5G,H). These results provide evidence that NP65 deficiency decreases Ribeye expression in the OPL of the retina. In addition, the post-synaptic proteins were explored in OPL by observing the distribution and expression of PKCα and calbindin (Figures 5I-K). PKCα immunostaining showed a slight increase in bipolar cell dendrites (Figure 5I, arrows indicated) in the OPL of NP65 KO mice, while no significant difference was observed in PKCα immunoreactivity (J) of the OPL between NP65 KO and WT mice. Also, the calbindin immunostaining showed that NP65 KO mice had a similar distribution (I) and comparable immunoreactivity (K) in OPL compared with WT mice. Taken together, these results conform the significant decrease in presynaptic structures PSD95, SYN and Ribeye in the OPL of NP65 KO mice, which contribute to the impairment of presynaptic transmission at the ribbon synapse and result in visual function impairment.

### NP65 KO mice show disarranged connections between photoreceptor cells and bipolar cells in OPL of retina

**FIGURE 6:**
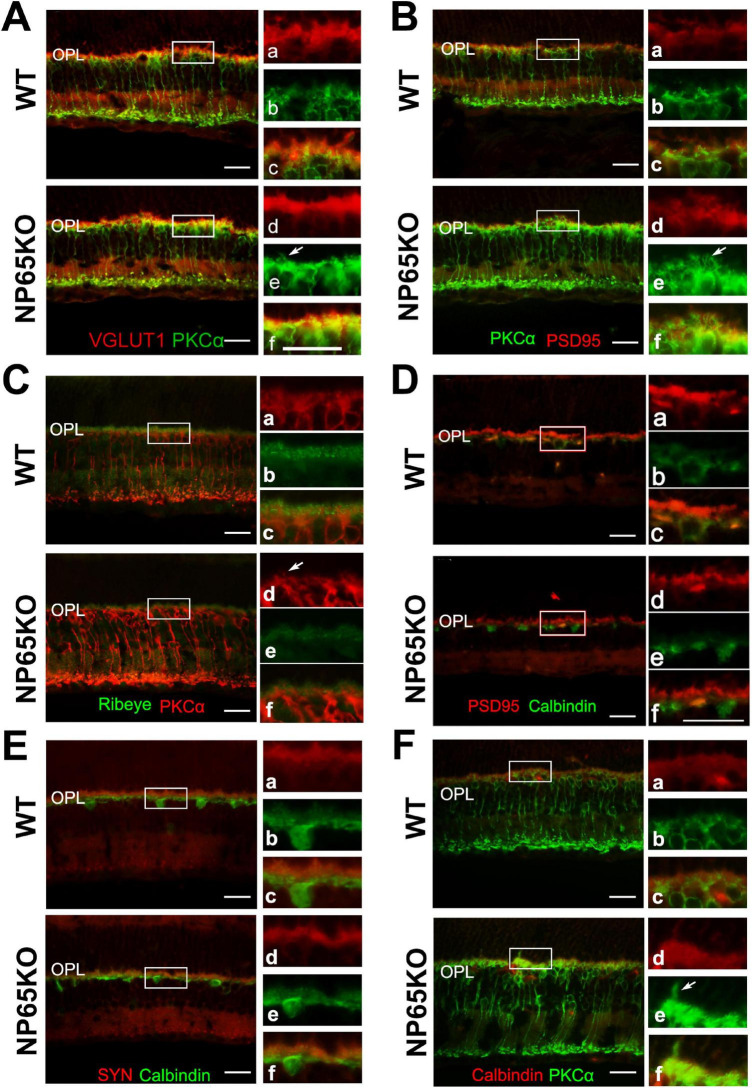
NP65 KO mice show disarranged connections between photoreceptor cells and bipolar cells in OPL of retina. **(A,B)** PKCα immunostaining also showed a slight increase in bipolar cell dendrites (A, B, arrows indicated) in the OPL of NP65 KO mice; VGLUT1/PKCα **(A)** and PSD95/PKCα **(B)** double immunostaining showed synaptic connection between photoreceptor and bipolar cells in the OPL of NP65 KO and WT mice. In the OPL of WT mice, the presynaptic terminals (red) labeled by VGLUT1 **(A)** or PSD95 **(B)** were closely juxtaposed to the bipolar cell dendrites (green) labeled by PKCα, only with few and faint yellow structures between the terminals (red) and dendrites (green) (**A,B**, and panel a–c); In the OPL of NP65 KO mice, there was some overlap or misplace between the presynaptic terminals(red) and the bipolar cell dendrites (green), showing more and obvious yellow structures between the terminals (red) and dendrites (green) (**A, B**, and panel **D–F**, disarrangement). **(C)** Ribeye/PKCα double immunostaining showed that Ribeye-positive puncta in NP65 KO mice were apparently decreased and were juxtaposed to PKCα-positive bipolar cell dendrites, similar to WT mice. **(D,E)** PSD95/calbindin **(D)** and SYN/calbindin **(E)** double immunostaining showed similar synaptic juxtaposition between photoreceptor and horizontal cells in the OPL of NP65 KO and WT mice. **(F**) PKCα/calbindin double immunostaining showed unchanged immunostaining and localization of horizontal cell tips and bipolar cell ends in the OPL of NP65KO mice compared with WT mice. The nucleus was stained with DAPI (blue). **(A-F)** Representative images are shown and observed on three sections derived from WT and NP65 KO mice (*n* = 3). Scale bars = 50 μm.

Next, we performed double immunostaining of PKCα with VGLUT1 or PSD 95 to determine if photoreceptor axon terminals are in close apposition to the dendrites of bipolar cells in the OPL. Consistent with Figure 5G, a slight increase in bipolar cell dendrites was observed in the OPL of NP65 KO mice (Figures 6A,B, arrows indicated). In the OPL of WT mice, the PSD95/PKCα and VGLUT1/PKCα double immunostaining showed that the presynaptic terminals (red) labeled by PSD95 or VGLUT1 were closely juxtaposed to the bipolar cell dendrites (green) labeled by PKCα, only with few and faint yellow structures between the terminals (red) and dendrites (green) (Figures 6A,B, and panel a-c), consistent with previous reports (Gong et al., 2006). However, in the OPL of NP65 KO mice, the presynaptic terminals (red) labeled by VGLUT1 or PSD95 appeared some overlap or misplace with the bipolar cell dendrites (green) labeled by PKCα, showing more and obvious yellow structures (Figures 6A,B, and D–F, disarrangement). This alteration indicates that the juxtaposition of presynaptic terminals labeled by VGLUT1 or PSD95 with dendrites labeled by PKCα may be somewhat disarranged in OPL of NP65KO mice (Figures 6A,B). Furthermore, ribbon synapses were also observed in the OPL by double immunostaining for Ribeye and PKCα. The merged images showed that Ribeye-positive puncta in NP65 KO mice were apparently decreased, consistent with Figures 5D,E, and were juxtaposed to PKCα-positive bipolar cell dendrites, as in WT mice (Figure 6C). Taken together, these results suggest that the synaptic connection between photoreceptor axon terminals and bipolar cell dendrites in the OPL may be misplaced in NP65 KO mice.

In addition, the synaptic connections between calbindin-positive horizontal cell tips and photoreceptor axon terminals (labeled by PSD95 or SYN) or bipolar cell dendrites (labeled by PKCα) in the OPL of NP65 KO mice were observed using double immunostaining. The merged observation showed that NP65 KO mice had similar immunoreactivity and apposition of PSD95/calbindin or SYN/calbindin and PKCα/calbindin in the OPL of the retina compared with WT mice (Figures 6D-F). These results suggest that synaptic connections in both photoreceptor/horizontal cells and bipolar cells/horizontal cells are unaffected in NP65 KO mice.

### Electron microscopy shows defects in ribbon synapse of OPL in NP65 KO mice

**FIGURE 7:**
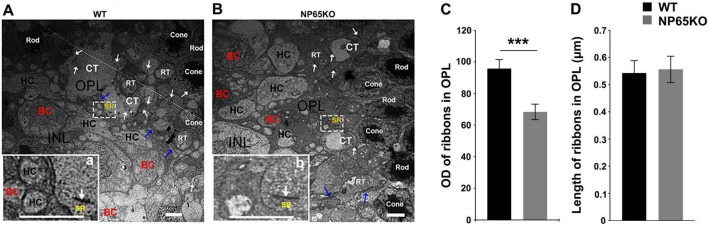
Electron microscopy shows defects in ribbon synapse in OPL of NP65 KO mice. **(A)** In the OPL of WT mice, a triad ribbon synapse was observed within a white rectangle. The enlargement (a) shows a synaptic ribbon (SR, arrow) in the cone axon terminals as well as two postsynaptic horizontal cell (HC) dendrites and an invaginating bipolar cell (BC) dendrite. **(B)** In the OPL of NP65 KO mice, a ribbon synapse was also observed within a white rectangle. However, the enlargement (b) shows a decrease in the electron dense deposit of the SR and a loss of dendrites. (**C**,**D)** Quantitative analysis displayed a significant reduction in optical density **(C)** and no difference in the length **(D)** of SR in the OPL of NP65 KO mice (*n* = 3). Rod, rod cell, RT, rod cell axon terminals; Cone, cone cell; CT, cone cell axon terminals. ***P < 0.001, Scale bar = 2 μm.

To further confirm the alteration of ribbon synapses in the OPL of NP65 KO mice, we performed ultra-thin slice transmission electron microscopy to observe the structures. As shown in Figure 7, in the OPL of WT mice, the triad ribbon synapse was observed, composed of synaptic ribbons in cone axon terminals and the postsynaptic structures of two horizontal cell (HC) dendrites and an invaginating cone bipolar cells dendrite (Figure 7A). In NP65 KO mice, the electron dense deposit of the synaptic ribbons was apparently decreased (Figures 7A-C), while the length of synaptic ribbons was no different compared with WT mice (Figures 7A,B,D). In addition, some postsynaptic structures were lost in the OPL in NP65 KO mice (Figures 7A,B). This defect of postsynaptic structures may reflect the disarrangement between the presynaptic photoreceptor axonal terminals and bipolar cell dendrites, which is consistent with that shown in Figure 6A. Therefore, the electron microscopy shows defects in ribbon synapse of the OPL in NP65 KO mice.

### Intravitreal injection of AAV-NP65 reverses visual dysfunction in NP65 KO mice

**FIGURE 8:**
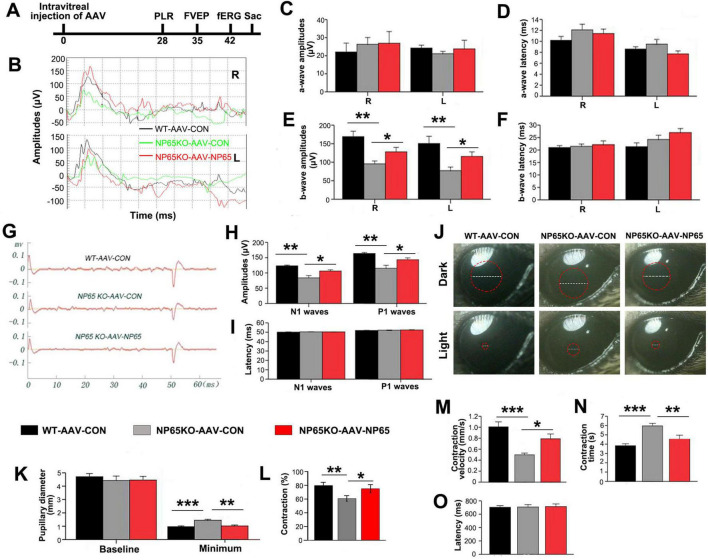
Intravitreous injection of AAV-NP65 reverses the impaired visual function in NP65 KO mice. **(A)** Timeline of PLR, fVEP, and fERG recordings after intravitreal injection. **(B–F)** In the scotopic flash electroretinogram (fERG), NP65 treatment reversed the decrease in b-wave amplitude in NP65 KO mice. **(B)** Representative fERG waveforms recorded from an NP65 KO and a WT mouse with AAV-NP65 or AAV-CON; **(C,D)** NP65 treatment did not affect the amplitude and latency of the a-wave in NP65 KO mice; **(E,F)** NP65 treatment reversed the decrease in b-wave amplitude with unchanged b-wave latency in NP65 KO mice. **(G–I)** In the flash visual evoked potentials (fVEP), NP65 injection reversed the reduction in amplitude of N1 and P1 in NP65 KO mice. **(G)** Representative fVEP waveforms from an NP65 KO and a WT mouse with AAV-NP65 or AAV-CON; **(H)** NP65 injection reversed the reduction in the amplitude of N1 and P1 in NP65 KO mice; **(I)** NP65 injection did not affect the latency of N1 and P1 in NP65 KO mice. **(J–O)** In the pupillary light reflexes (PLR), NP65 treatment prevented a decrease in contraction rate and contraction velocity in NP65 KO mice; **(J)** Representative pupil images from an NP65 KO and a WT mouse with AAV-NP65 or AAV-CON; **(K)** NP65 treatment had no effect on the baseline pupillary diameter while decreasing the minimum pupillary diameter in NP65 KO mice; **(L,M)** NP65 treatment enhanced the constriction **(L)** and velocity **(M)** of the pupil in NP65 KO mice; **(N,O)** NP65 treatment decreased the constriction time with unchanged latency of constriction in NP65 KO mice. *n* = 8-9, *P < 0.05, **P < 0.01, ***P < 0.001.

To further confirm the hypothesis that NP65 deficiency leads to impaired visual function, we administered AAV-NP65 to the retina of NP65 KO mice by intravitreal injection. Four weeks after viral injection, a series of visual function tests were performed as above. As shown in Figure 8, NP65 KO mice treated with AAV-CON (NP65 KO control) exhibited impaired responses in fERG, fVEP and PLR compared with WT mice treated with AAV-CON (WT control), consistent with the results in Figure 1. As expected, AAV-NP65 treatment reversed the decrease in b-wave amplitude in fERG (Figures 8B-F), N1 and P1 amplitude in fVEP (Figures 8G-I) and contraction rate and contraction velocity of PLR (Figures 8J-O) in NP65 KO mice. Therefore, these results demonstrate that the administration of AAV-NP65 into the retina can restore the visual function in NP 65 KO mice, suggesting that NP65 is required for the normal visual function of mice.

**FIGURE 9:**
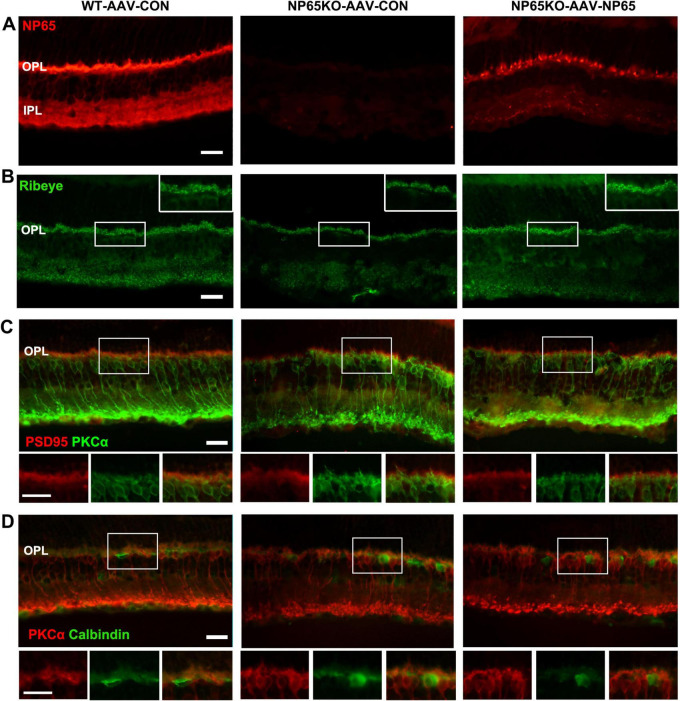
Intravitreous injection of AAV-NP65 exhibits the increase of ribbon synapse and restore arrangement in OPL of NP65 KO mice. **(A)** NP65 immunostaining showed that NP65 expression in the retina of NP65 KO and WT mice with AAV-NP65 or AAV-CON. Notably, there was prominent NP65 expression in the OPL of NP65 KO-AAV-NP65 mice. **(B)** Ribeye immunostaining showed that administration of AAV-NP65 significantly increased the number of Ribeye-positive puncta in the OPL of the NP65 KO mice. **(C)** Double immunostaining of PSD95/PKCα revealed that NP65 supplementation partially restored the normal arrangement of PSD95/PKCα connectivity in the OPL of NP65 KO mice. **(D)** Double immunostaining of PKCα/calbindin showed that NP65 replenishment had no effect on the immunoreactivity and apposition of PKCα/calbindin. **(A–D)** Representative images are shown and observed on three sections derived from WT and NP65 KO mice with viral injection (*n* = 3), Scale bars = 20 μm.

After the test of visual function, the retina was collected to measure NP65 levels and retinal structures. As shown in Figure 9A, AAV-NP65 intravitreous injection caused a significant increase in NP65 expression in the inner half of the retina in NP65 KO mice. Importantly, NP65 was abundantly located in the OPL of NP65 KO mice while the level of NP65 in NP65 KO mice was still lower compared with that of WT mice. To determine if NP65 replenishment affects synaptic ribbons in the OPL, Ribeye expression was evaluated in the retina after AAV-NP65 treatment. Consistent with the above results (Figures 5E,F, 6C), NP65 KO control showed a decrease in Ribeye-positive puncta in the OPL compared with that of WT mice (Figures 9B). Interestingly, administration of AAV-NP65 exhibited an increase in Ribeye-positive puncta in the OPL of NP65 KO mice (Figure 9B). Combined with the results in Figures 5E-I, 6C, 7, these findings demonstrate for the first time that NP65 regulates the Ribeye expression of ribbon synapse in the OPL. Next, the synaptic connection of photoreceptor/bipolar cells and bipolar/horizontal cells in the OPL was explored and identified by double immunolabeling of PSD95/PKCα and PKCα/calbindin. We found that the positive structures of PSD95/PKCα were juxtaposed in the OPL of NP65 KO control mice, similar to the pattern in Figure 6B. However, the NP65 replenishment partly restored the normal arrangement of PSD95/PKCα synaptic connection in the OPL of NP65 KO mice (Figure 9C). NP65 replenishment had no effect on the immunoreactivity and apposition of PKCα/calbindin (Figure 9D). Therefore, NP65 replenishment in the OPL can increase the Ribeye expression and restore the arrangement in the OPL, which underlies the reversal of visual dysfunction in NP65 KO mice.

## Discussion

In this study, we have shown that NP65 KO mice exhibit impaired visual function. NP65 KO mice showed a reduction in ribbon synapses and disarrangement between photoreceptor axon terminals and bipolar cell dendrites in the OPL. Vitreous injection of AAV-NP65 alleviated the impairment of visual function and increased ribbon synapses and restoring a normal arrangement in the OPL in NP65 KO mice. Our results identify for the first time that NP65 is required for normal visual function in mice.

Despite the fact that NP65 is shown to be expressed in the synaptic layers of the rat and mouse retina (Kreutz et al., 2001; Carrott et al., 2016), the role of NP65 in visual function is still unknown. In our present study, NP65-deficient mice displayed a significantly reduced amplitude of the b-wave in fERG, a lower amplitude of N1 and P1 waves in fVEP, and reduced constriction in PLR. Therefore, we clearly showed that NP65 is crucial for normal visual function in mice. Similarly, Basigin, another member of the NCAM, has been shown to be crucial for retinal function, with Basigin knock-out mice exhibiting impaired visual function (Ochrietor et al., 2002). Nevertheless, Carrott et al. assumed that both isoforms of Nptn have non-essential roles in the mouse retina, based on the observation that ENU-generated Nptn mutation mice (lacking both NP65 and NP55) showed normal retinal function, as assessed using ERG (Carrott et al., 2016). However, because ENU-generated Nptn variants may express altered or truncated neuroplastin proteins, the underlying mechanisms could differ from our targeted NP65-deficient mice which lacks neuroplastin 65 entirely while NP55 remains intact (Amuti et al., 2016).

In contrast to previous reports showing high NP65 immunoreactivity confined to the OPL and IPL (Kreutz et al., 2001; Carrott et al., 2016), we found that NP65 was localized throughout all layers of the mouse retina, including the RPE, ONL, OPL, INL, IPL, and GCL. It should be noted that the Kreutz study did not mention NP65 immunostaining in the ONL (Kreutz et al., 2001). In our study, the most intense immunostaining was found in the OPL, while the faintest was in the GCL. NP65 immunostaining was also observed in the processes of the ONL and INL. Double immunostaining of NP65/VGLUT1, SYN, or PSD and NP65/PKCa or calbindin clearly showed that NP65 is expressed in both presynaptic (axonal terminals of the photoreceptors) and postsynaptic (the dendrites of bipolar cells and horizontal cells) regions in the OPL. This is consistent with the findings of Kreutz et al., who also showed that NP65 is localized in pre- and postsynaptic regions in the OPL through *in situ* hybridization and NP65/VGLUT1 immunoreactivity (Kreutz et al., 2001). Importantly, we found that Ribeye-positive puncta completely colocalized with NP65 immunoreactivity in the OPL and IPL, suggesting a role for NP65 in ribbon synapses. Therefore, the widespread expression of NP65 throughout the layers of the retina and its specific localization in ribbon synapses suggests its involvement in visual function.

Indeed, we found that NP65 KO mice exhibited impaired vision function despite having nearly normal histological structures in the retina, including the numbers of neurons in ONL, INL, and GCL, as well as astrocytes and Müller cells, compared with WT mice. Given that the most intense NP65 immunoreactivity localized in the OPL and the reduction in amplitude of the b-wave in fERG, and the reported function of neuroplastins in synaptogenesis (Beesley et al., 2014; Herrera-Molina et al., 2014), we explored the possibility that the OPL alteration in NP65 KO mice contributes to vision dysfunction. As expected, the immunoreactivity of SYN and PSD95 was significantly reduced in NP65 KO mice. Most importantly, we found that both the Ribeye immunoreactivity and the electron dense deposit of the synaptic ribbon were apparently decreased in NP65 KO mice compared to WT mice. These results show that the presynaptic decrease in the OPL in NP65 KO mice may be related to impaired vision function, specifically indicated by the reduction in amplitude of the b-wave in fERG. Furthermore, the delivery of AAV-NP65 in the retina reversed the visual dysfunction and increased Ribeye immunoreactivity in NP65 KO mice. Consistent with these findings, previous reports have shown that Ribeye is essential for synaptic ribbon organization and presynaptic neurotransmitter release (Maxeiner et al., 2016; Shankhwar et al., 2022). In addition, Ribeye KO mice exhibited a reduced ERG b-wave amplitude and an unchanged a-wave amplitude (Fairless et al., 2020; Mesnard et al., 2022). Thus, our results demonstrate that NP65 is critical for OPL ribbon synapse formation and retinal function in mice.

At all stages, at least some of the synapses have appropriate pre- and postsynaptic molecular constituents that are juxtaposed, indicating apparently intact synapses. Recently, it has been shown that, in hippocampal cultures from Nptn-deficient mice (loss of NP65 and NP55), the number of newly formed glutamatergic (excitatory) synapses was unchanged but there was an increased disarrangement between presynaptic and postsynaptic terminals compared with wild-type-derived cultures (Herrera-Molina et al., 2014). However, with maturation, the number of excitatory synapses was reduced in Nptn-deficient cultures. These morphological defects were thought to be linked to the absence of NP65, but not NP55, and were likely a result of synaptic instability caused by the loss of trans-homodimerization via the NP65-specific Ig1 domain (Herrera-Molina et al., 2014). In addition, an ENU-generated Nptn mutation mouse was found to have incorrectly juxtaposed ribbon synapses of inner hair cells, with an increased disarrangement between presynaptic ribbons (Ribeye) and postsynaptic densities compared with wild-type mice (Carrott et al., 2016). Typically, synaptic CAMs span the synaptic cleft through homophilic or heterophilic interactions and are anchored to distinct cytoskeletal elements on either side of the synapse (Beesley et al., 2014; Owczarek and Berezin, 2012). They are thus well placed to mediate synapse formation and stabilization during development. Given the fact that NP65 is present at pre- and postsynaptic regions in the OPL and may potentially engage in trans-homophilic binding in the synaptic cleft (Smalla et al., 2000; Owczarek et al., 2011), it is possible that some photoreceptor axon terminals and bipolar cell dendrites are misplaced in the absence of NP65. In fact, double staining of VGLUT1 or PSD95 with PKCα showed that there were disarrangements between the axon terminals of photoreceptor and the dendrites of bipolar cells in the OPL. Electron microscopy observation showed a defect of a triad ribbon synapse in the OPL of NP65 KO mice. It is well known that an impaired amplitude of the b-wave in fERG represents impaired synaptic transmission in the OPL (Mdzomba et al., 2018). Thus, it is supposed that the disarrangements in the OPL contributes to the impaired transmission, which consequently leads to impaired b-waves in ERG in NP65 KO mice. Moreover, our delivery of AAV-NP65 in the retina reversed the impaired visual function with restored normal apposition of PSD95/PKCα immunosignals in the OPL, providing direct evidence that the loss of NP65 affects synapse formation in the OPL and results in impaired visual function. In addition, since fVEP represents the pathway from the retina to the visual cortex and PLR indicates the reflex arc from the retina to the pupillary sphincter, it needs to be determined whether other parts of the pathway except the retina are abnormal in the near future.

In conclusion, we have uncovered that Neuroplastin 65 is required for ribbon synapses in the OPL, which are a prerequisite for normal visual function.

## Data Availability

The original contributions presented in the study are included in the article/supplementary material, further inquiries can be directed to the corresponding author.
